# Video-Based Hand Movement Analysis of Parkinson Patients before and after Medication Using High-Frame-Rate Videos and MediaPipe

**DOI:** 10.3390/s22207992

**Published:** 2022-10-20

**Authors:** Gökhan Güney, Talisa S. Jansen, Sebastian Dill, Jörg B. Schulz, Manuel Dafotakis, Christoph Hoog Antink, Anne K. Braczynski

**Affiliations:** 1 KIS*MED (AI Systems in Medicine), Technische Universität Darmstadt, Merckstraße 25, 64283 Darmstadt, Germany; 2 Department of Neurology, RWTH University Hospital, 52074 Aachen, Germany; 3 Jülich Aachen Research Alliance (JARA)–JARA-Institute Molecular Neuroscience and Neuroimaging, FZ Jülich and RWTH University, 52428 Jülich, Germany; 4 Institut für Physikalische Biologie, Düsseldorf, Heinrich-Heine University, 40225 Düsseldorf, Germany

**Keywords:** Parkinson’s disease, tremor, video-based analyses, mediapipe, artificial intelligence

## Abstract

Tremor is one of the common symptoms of Parkinson’s disease (PD). Thanks to the recent evolution of digital technologies, monitoring of PD patients’ hand movements employing contactless methods gained momentum. **Objective:** We aimed to quantitatively assess hand movements in patients suffering from PD using the artificial intelligence (AI)-based hand-tracking technologies of MediaPipe. **Method:** High-frame-rate videos and accelerometer data were recorded from 11 PD patients, two of whom showed classical Parkinsonian-type tremor. In the OFF-state and 30 Minutes after taking their standard oral medication (ON-state), video recordings were obtained. First, we investigated the frequency and amplitude relationship between the video and accelerometer data. Then, we focused on quantifying the effect of taking standard oral treatments. **Results:** The data extracted from the video correlated well with the accelerometer-based measurement system. Our video-based approach identified the tremor frequency with a small error rate (mean absolute error 0.229 (±0.174) Hz) and an amplitude with a high correlation. The frequency and amplitude of the hand movement before and after medication in PD patients undergoing medication differ. PD Patients experienced a decrease in the mean value for frequency from 2.012 (±1.385) Hz to 1.526 (±1.007) Hz and in the mean value for amplitude from 8.167 (±15.687) a.u. to 4.033 (±5.671) a.u. **Conclusions:** Our work achieved an automatic estimation of the movement frequency, including the tremor frequency with a low error rate, and to the best of our knowledge, this is the first paper that presents automated tremor analysis before/after medication in PD, in particular using high-frame-rate video data.

## 1. Introduction

Parkinson’s disease (PD) is one of the most frequent movement disorders that was first described by James Parkinson in 1817 [1]. This disorder severely affects the patient’s quality of life [2]. Worldwide, more than six million individuals suffer from PD [3]. Three out of four PD patients develop a tremor [4]. Clinical evaluation of PD is generally done qualitatively or semi-quantitatively using validated scales, like the Unified Parkinson’s Disease Rating Scale (UPDRS) [5]. Quantitative methods, such as electromyography optionally, including an accelerometer, are time-consuming and sensitive to any other body movement that corrupts the signal [6]. In this context, research on automated diagnosis may have significant importance in assisting medical professionals in the diagnosis and monitoring of tremors.

Due to the evolution of digital technologies, alongside the improved capabilities of machine learning algorithms, we observe a strong increase in research activity regarding automatic monitoring of PD motor symptoms [7], monitoring of the hands being of particular interest [8]. In this context, researchers have mostly focused on smart devices, such as smartwatches [9,10,11] and smartphones [12,13,14]. However, in recent years, non-contact sensing modalities using cameras have emerged with tremendous success [15]. These modalities could complement or even replace some of the existing contact-based technologies soon since they offer several advantages. Contactless measurements provide more comfortable diagnostics for patients [16], are more hygienic, and are easier to set up. Research on how to utilize video recordings to monitor PD motor symptoms has been performed in several works [17,18,19,20,21].

In the literature, researchers have presented different approaches for video-based analysis. Uhríková et al. [17] extracted signals based on intensity changes between video frames (25 fps) for manually marked hands and conducted frequency analysis. Pintea et al. [18] proposed Lagrangian and Eulerian approaches for hand-tremor frequency estimation from videos (30 fps) localizing the hand by Convolutional Pose Machines [22]. To improve the accuracy of Kinect-based (30 fps) estimation of amplitude and frequency, Alper et al. [19] presented a fusion of Pose and Optical Flow methods analyzing a specified region of interest (ROI). In another study, Williams et al. [20] used a smartphone to record videos (60 fps) and performed the frequency analysis by manually localizing the hand.

The aforementioned studies showed the general feasibility of the approach but were limited by the need for pre-processing, manual labeling, cropping, model training, and/or ROI selection. An approach using automatic hand or hand region detection without model training could facilitate the usage of video-based hand movement/tremor analysis methods and shorten the analysis time by eliminating the labeling, algorithm training, or ROI selection process.

In the following work, we performed an automatic video-based analysis of hand movement using high-frame-rate (≥180 fps) video recordings with artificial intelligence (AI)-based hand-tracking technology for before- and after-medication cases. We used the novel MediaPipe [23] framework to detect the hand and derive location changes between video frames automatically. Firstly, we compared the estimation of frequencies and amplitudes from the video and the accelerometer data to show the accuracy of our approach. In the second step, we investigated the frequency and amplitude changes before (OFF-state) and after medication in patients undergoing standard oral treatment, including L-Dopa and dopamine agonists (ON-state). To the best of our knowledge, this is the first paper that presents automated analysis of hand movements of patients suffering from PD before and after medication with a contactless measurement method, in particular using high-frame-rate video data.

## 2. Materials and Methods

In this section, we describe the data recording and processing process.

### 2.1. Participants

In this study, 11 PD patients (between 52 and 83 years of age, mean age 66 years, 7 males and 4 females) participated. On average, they had been diagnosed with PD for 10 years (min. 3, max. 20 years). In terms of disease severity, the Hoehn and Yahr classification was between 2 and 3, and the average UPDRS Part III was 37.9 points (min. 22, max. 73 points). All patients were in-house patients of university hospital Aachen enrolled in a 3-week rehabilitation program. The accelerometer data was recorded before medication, and videos of the affected hand (in pronation position) were recorded before and after medication. Extended information, including the UPDRS on patients, is given in Table 1. Question 3.15 of the UPDRS estimates the postural tremor of the hands, only the tremor-dominant site is given. The rating is between 0 (normal) and 4 (severe) [5]. Medication was taken according to the regular, individual scheme, the measurements were done just before the next regular medication intake and then 30 min after.

### 2.2. Accelerometer Data Recording

Accelerometer data were recorded for 30 s with the Natus Nicolet™ VikingQuest EMG/EP (Natus, Middleton, WI, USA) equipment by the same researcher throughout the study with a standardized methodology. The subjects were sitting in a quiet temperature-controlled room. An acceleration sensor was placed on the middle phalanx of the index finger of the upper extremity of the tremor-dominant side while holding the hand still. Surface-recording EMG electrodes were placed on the wrist (data not used). The raw data of the recorded curves were then transferred to Matlab (Mathworks, Natick, MA, USA) for further analysis.

### 2.3. Video Recording

The videos were taken of the tremor-dominant hand for 30 s with a camera, Lumix GH5, Kadoma, Japan (PAT001 and PAT002), GoPro HERO7, San Mateo, CA, USA (all others). The recording settings were set to slow-motion video recording with at least 180 frames/s. Image processing was performed with Python (Python Software Foundation) and Matlab (Mathworks, Natick, MA, USA).

### 2.4. Data Extraction and Processing

**Figure 1:**
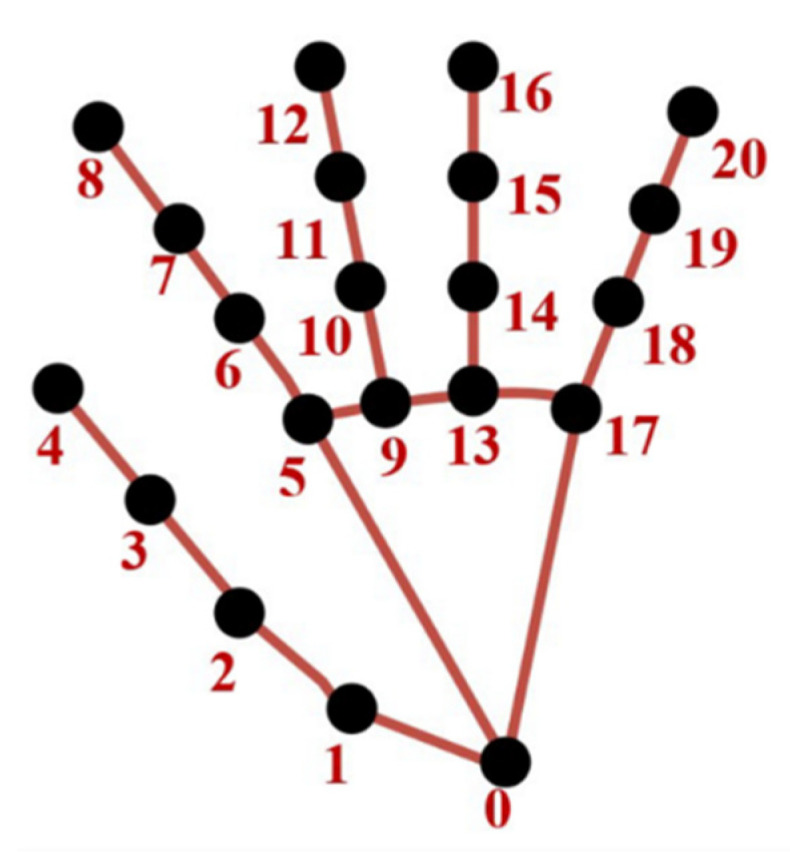
MediaPipe hand landmarks.

The main data extraction and processing concept in this work aims to track the hand movements from video recordings with high accuracy and precision. We used the AI-based novel MediaPipe [23] hands algorithm to extract hand location changes automatically from the videos. To achieve an accurate measurement, captured images should not contain motion blur, which can be achieved by employing high-frame-rate cameras. MediaPipe uses two models in a pipeline (a hand palm detection model and a hand landmark model). Each frame of a video is fed into the palm detection model, which produces a bounding box based on the palm. The hand landmark model uses this bounding box as an input and returns a list of 21 hand landmarks given in Figure 1, each of which has three coordinates (x, y, and z). Detailed descriptions of landmarks are given in Table 2. The frame width and height, respectively, are used to normalize x (width) and y (height) to [0.0, 1.0]. z denotes the landmark depth. By multiplying x with the frame width and y with the frame height, the vertical and horizontal pixel coordinates of the hand landmarks can be calculated.

**Figure 2:**
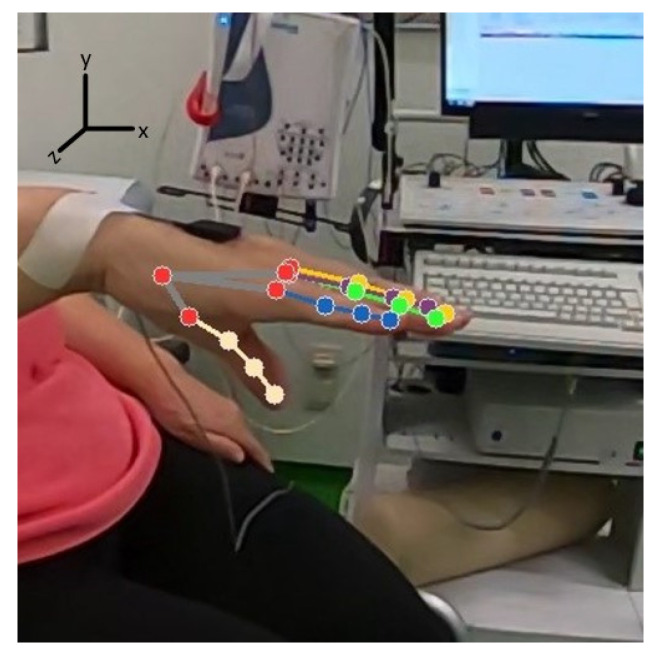
Example landmarks extraction process (beige: thumb, lilac: index finger, yellow: middle finger, green: ring finger, blue: little finger, red: wrist, carpometacarpal and metacarpophalangeal joints).

Depending on the hand positions (some patients start keeping their hand on their lap and then lift it later on, or some put it down before the video ends), a couple of seconds from the start and end of every video were cut. From the remaining videos, 21 landmarks of the affected hand were extracted sequentially over 10 s and represented as a 10-sec long signal. Besides blur-free videos, using a high-frame-rate helps to determine the correct signal amplitude and waveform of the extracted one-dimensional signal, as its sampling frequency obviously corresponds to the frame-rate. A sample landmarks extraction is given in Figure 2.

Since videos were recorded from a viewpoint where vertical hand movement is easy to observe, the information from the vertical (y) axis of the hand landmarks was used for video-based acceleration estimation. Both the reference accelerometer data and extracted video time-series data were band-pass filtered between 0.5–15 Hz.

Generally, variations of frequency over time in tremors are expected. To observe these changes, following the filtering, both accelerometer and video time-series data (for all landmarks) were segmented into non-overlapping 2-s long signals (15 segments of 2-s signal for accelerometer and 5 segments of 2-s signal for video data). The segment length of 2 s was chosen to obtain a more detailed and precise frequency spectrum in comparison to 1-s segment length.

**Figure 3:**
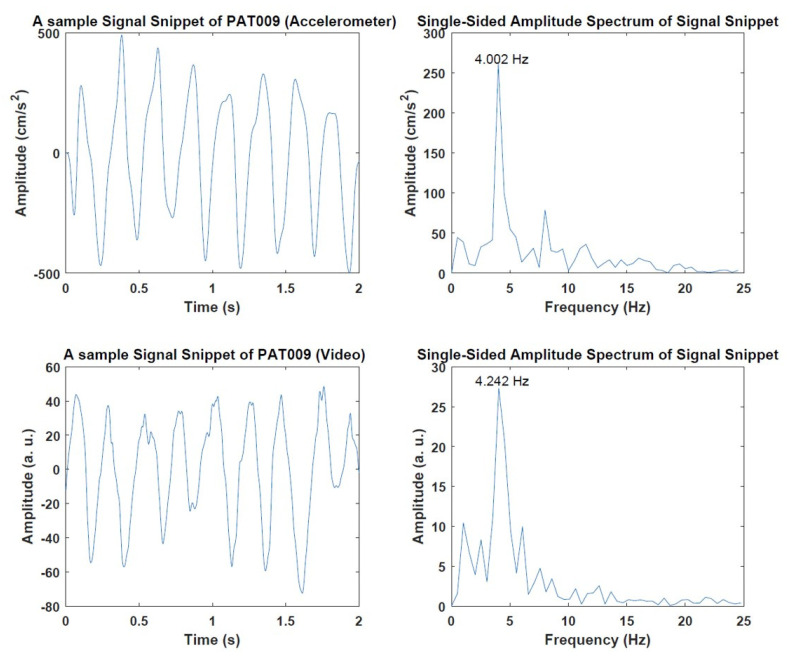
Example signals from PAT009. Accelerometer signal (top-left) and corresponding calculated frequency (top-right). Signal representation of wrist landmark (bottom-left) and corresponding estimated frequency (bottom-right).

In the next step, all segmented data are converted into the frequency domain using the Fast Fourier Transform (FFT). Dominant frequency calculation from the accelerometer and estimation from video data were conducted as follows for every patient. Samples of accelerometer and video time-series data with FFT calculations are given in Figure 3.

For the accelerometer data, the mean frequency value of 15 signal snippets was calculated. Since the goal of this work is to find only the dominant tremor frequency throughout the experiment, frequency values that deviated more than three standard deviations from the mean were considered outlier values and were eliminated automatically. To have a robust calculation of the dominant accelerometer frequency, the rest of the values were averaged, and the averaged value was noted as the dominant frequency and used as a baseline to compare the camera-based approach.

**Figure 4:**
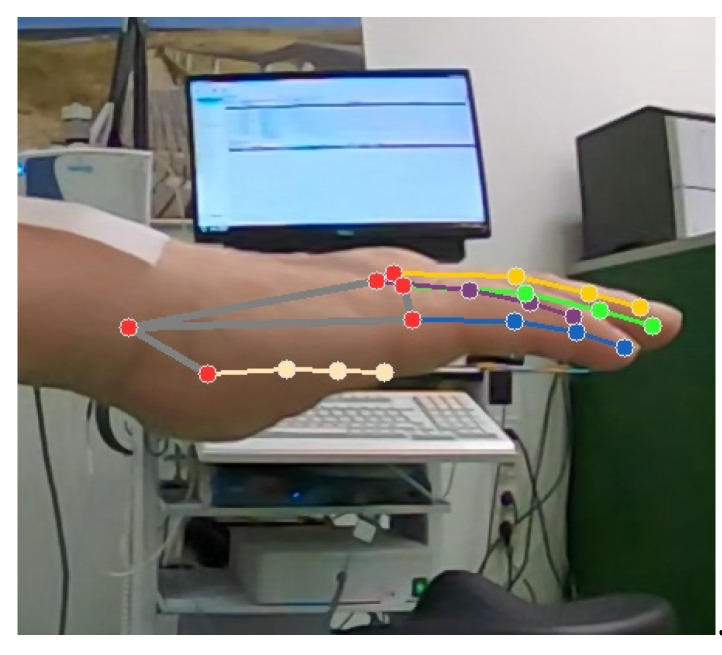
Example landmarks extraction process (the thumb is not visible in the video, but landmark positions are estimated (beige: thumb, lilac: index finger, yellow: middle finger, green: ring finger, blue: little finger, red: wrist, carpometacarpal and metacarpophalangeal joints).

During the frequency estimation from video data, some landmark points produced frequencies with a high deviation from the real accelerometer frequency (see Section 3.1). The most important reason for these outliers is that not all landmarks estimated are actually visible in the video at all times because of hand position and resulting occlusion. For this situation, MediaPipe works based on a position assumption considering other landmarks and creates pseudo/noisy data of those landmarks. A visualization of this is given in Figure 4.

Data points responsible for the outlier frequency values were excluded automatically, as described above for the accelerometer data. Additionally, to suppress the effect of pseudo/noisy landmark values and to obtain a robust frequency estimation, frequencies from the (non-excluded) landmarks were averaged, and the dominant frequency was calculated. The same method was applied both before- and after-medication. Besides frequency analysis, the amplitude analysis was also conducted by averaging the amplitudes of the maximum frequencies from the power spectrum to calculate the amplitude.

## 3. Results

This section presents the results of the frequency and amplitude estimations. First, ground-truth accelerometer data and data extracted from the videos were compared (before medication). Second, results were analyzed for before and after-medication cases (video only).

### 3.1. Preliminary Results of Video and Accelerometer Data

**Figure 5:**
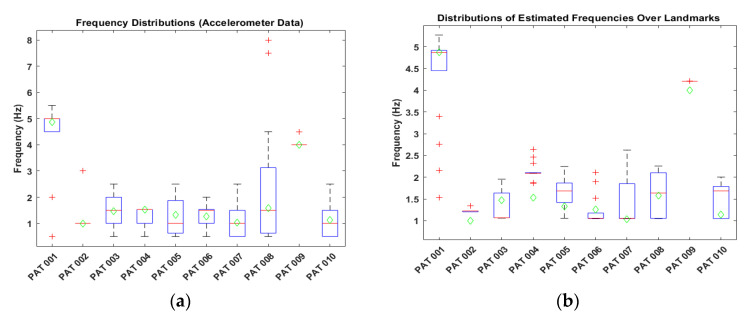
Frequency distributions. (**a**) Distribution of the accelerometer signals (15 frequency calculations were used for each patient). (**b**) Distribution of the video data over all landmarks (frequency calculations of 21 landmarks were used for each patient). Green diamonds correspond to the accelerometer’s calculated dominant frequency.

In this part, the frequency distributions for the accelerometer and video data are visualized before the outlier elimination process. Figure 5 shows the distributions of estimated frequencies.

From Figure 5, we see that both accelerometer and video data contain some outlier values which need to be eliminated to have a robust frequency calculation and estimation.

### 3.2. Video and AccelerometerAnalysis Results

A comparison of dominant frequencies between accelerometer and video data is provided in Table 3. Results were compared using Absolute Error (AE). The formula of the AE can be expressed as follows:

(1) $$\Delta f=\left|f_{\mathrm{acc}}-{\;f}_{\mathrm{vid}}\right|,$$

where $f_{\mathrm{acc}}$ denotes accelerometer frequency, and $f_{\mathrm{vid}}$ denotes frequency extracted from the video. In addition to AE, the Root Mean Square Error (RMSE) between frequencies of the accelerometer and video data was calculated as:

(2) $$\mathrm{RMSE}=\sqrt{\frac{1}{n}\overset{n}{\underset{i=1}{\sum }}{\left(f_{\mathrm{acc},i}-{\;f}_{\mathrm{vid},i}\right)}^{2}}$$

where n denotes the number of patients. Table 3 presents the estimated dominant frequencies.

**Figure 6:**
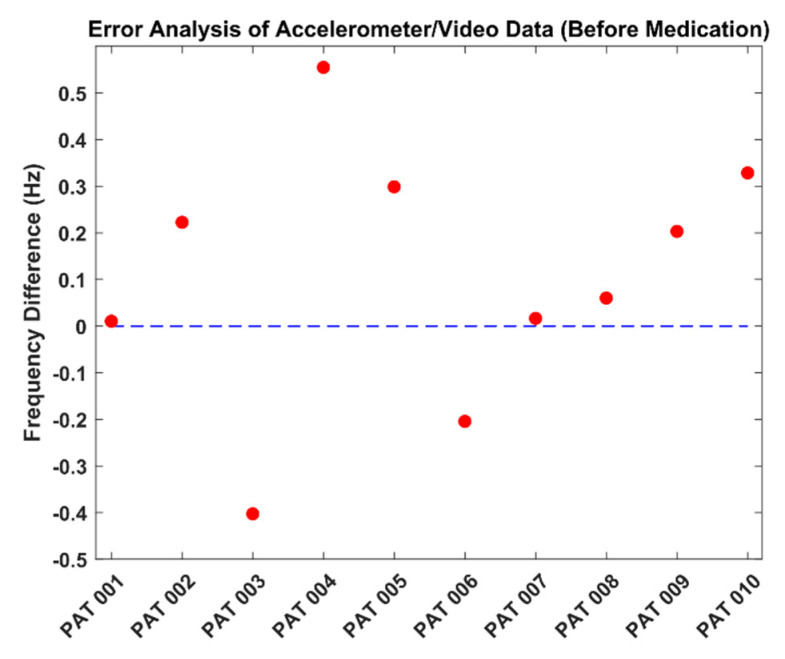
Frequency error analysis (analysis was performed in terms of dominant frequency for all patients).

For PAT011, no accelerometer data was available. In Table 3, the highest error was found at 0.554 Hz, while the lowest error was 0.011 Hz. RMSE was calculated as 0.283 Hz. Figure 6 presents the error analysis between the accelerometer and video data.

From Figure 6, we see that the frequency values were estimated with small deviations. In ten patients, only the frequencies of two patients were estimated to be lower than the accelerometer frequency (PAT 003 and PAT 006).

**Figure 7:**
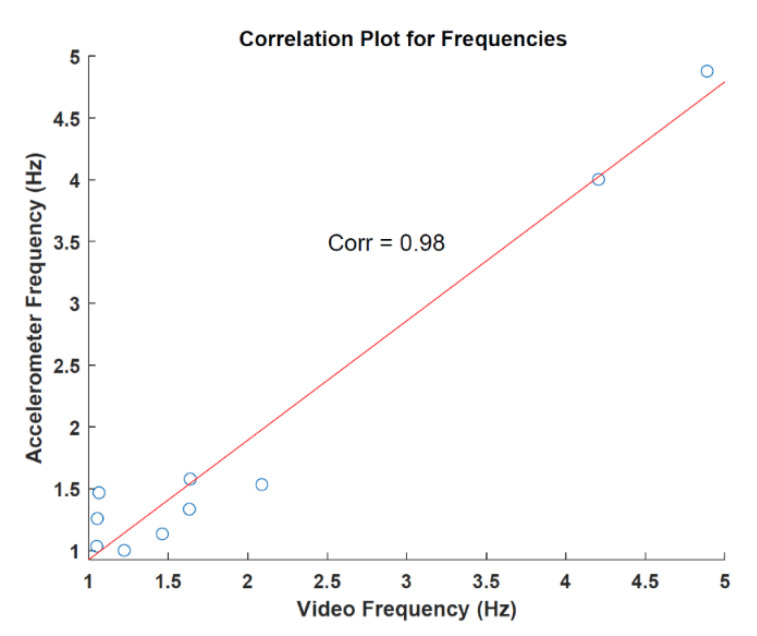
Correlation analysis for frequencies.

To observe the relation between the accelerometer and data extracted from the videos, the correlation was calculated over the respective frequencies. The correlation was found to be 0.98 with a *p*-value of 7.256 × 10^−7^. Figure 7 shows the result of the analysis. In terms of tremor analysis, two patients showed the classical parkinsonian tremor with 4 Hz (PAT 001 and PAT 009) detectable in both accelerometer and video data. This tremor was clinically present and represented in the UDPRS part 3.15 (score ≥ 2). All other patients showed a lower rhythmic movement with detectable frequencies of 1–2 Hz. From the clinical evaluation, there was no Parkinsonian tremor as measured via the UPDRS (see Table 1). The possible reasons for this rhythmic movement are discussed below.

In addition to frequency calculations, amplitudes were also estimated. Results of the estimated amplitude of the accelerometer and video data are given in Table 4.

**Figure 8:**
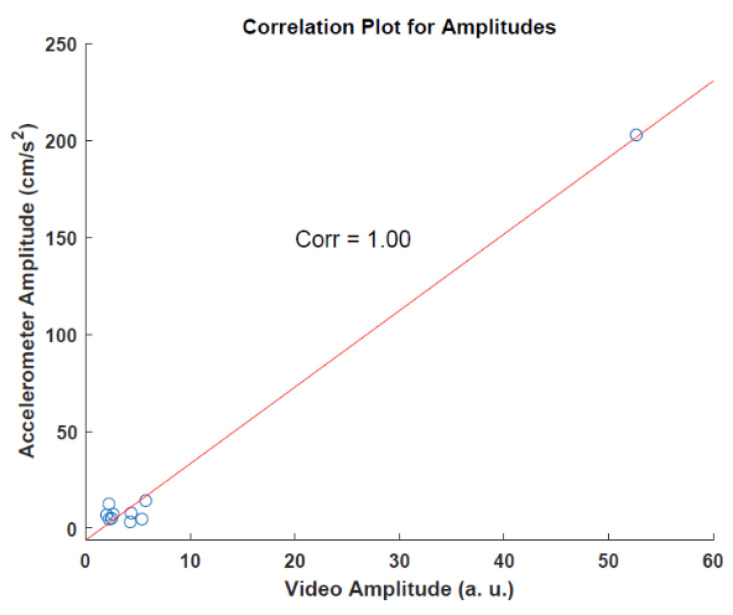
Correlation analysis for amplitudes.

Since the two data sources use different scales and units, extracted amplitudes cannot be compared directly. However, to observe the association between these methods, the correlation was calculated for the amplitudes. For the correlation analysis of amplitudes, we found a correlation of 1.0 with a *p*-value of 2.041 × 10^−9^. Figure 8 shows the result of the analysis.

### 3.3. Video before/after Medication Analysis Results

In this study, the video-based examination of the medication effect was also explored. Table 5 provides the estimated dominant frequencies and changes before and after medication for the video data. Frequency changes were calculated by subtracting the before-medication frequency from the after-medication frequency.

**Figure 9:**
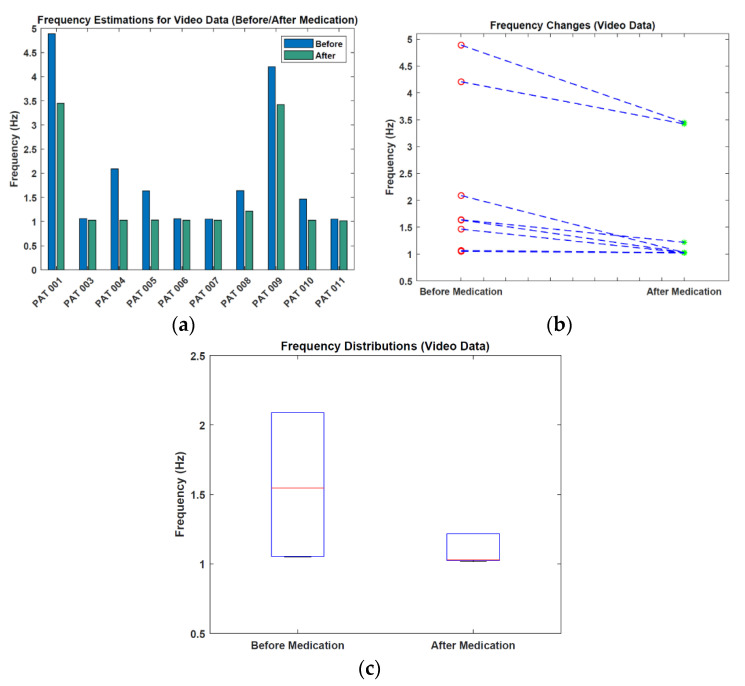
Visualization of the frequency results. (**a**) Frequency estimations. (**b**) Frequency changes. (**c**) Frequency distributions (values > 3 Hz are not shown).

In Table 5, all patients had a decreased frequency value for the after-medication case compared to the before-medication case. The mean frequency value decreased by 0.487 Hz after medication. Figure 9 shows the visualization of frequency estimations and changes.

From Figure 9, we see that the median frequency of 1.5 Hz before medication decreases by approximately 0.5 Hz after medication. Moreover, the spread of the frequencies decreased.

As before, we also performed an amplitude analysis additional to the frequency analysis to see the changes before and after medication. Estimated amplitudes and changes for before and after medication for the video data are given in Table 6. Amplitude changes were calculated by subtracting the before-medication amplitude from the after-medication amplitude.

**Figure 10:**
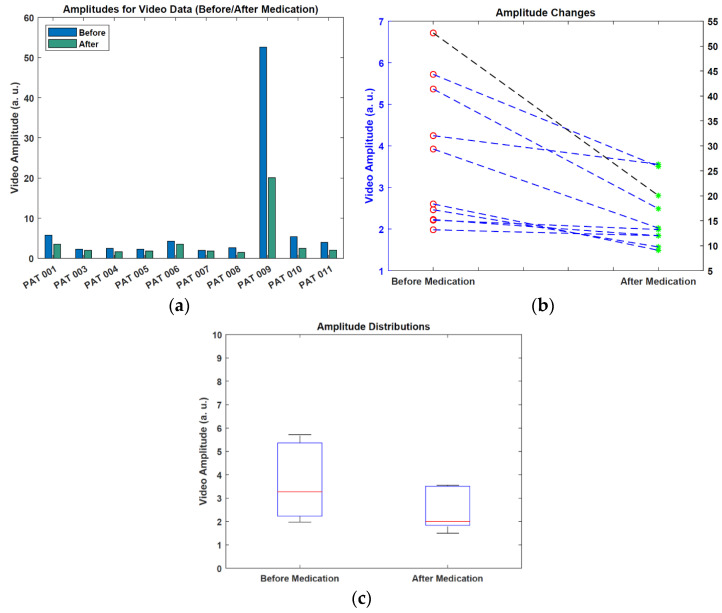
Visualization of the amplitude results. (**a**) Amplitude estimations. (**b**) Amplitude changes (note the different scale for one data point). (**c**) Amplitude distributions (values > 10 a.u. are not shown).

In Table 6, all patients had a decreased amplitude value for the after-medication case compared to the before-medication case. The mean amplitude value dropped from 8.167 a.u. to 4.033 a.u after medication. In general, for the patients that have minor frequency changes after medication, amplitude changes were also small (under 1 a.u.). Figure 10 shows the visualization of amplitude estimations and changes.

From Figure 10, we can see that some patients exhibited only minor changes in the amplitude, however, the amplitude distribution graph shows that there is a noticeable amplitude change between the two cases.

## 4. Discussion

In this study, our aim was a video-based analysis of tremors using high-frequency video recordings and an AI-based hand-tracking approach. To evaluate the accuracy of the video-based method, the Absolute Error between frequencies extracted with the accelerometer and with the video analysis was calculated. As seen in Table 3, the highest error was calculated as 0.554 Hz, while the lowest error was 0.011 Hz (mean = 0.229 Hz). In addition to this low absolute error, the correlation between accelerometer and video was very high (0.98). Second, amplitudes from the power spectrum were calculated for both video and accelerometer data. Since these two methodologies have different scales and units, a direct comparison of them is not possible. Instead, we calculated the correlation. The result showed a high correlation between amplitudes, which led us to use our amplitude measurement approach in the next step. We acknowledge that the number of subjects is small, and thus, the high correlation has to be taken with a grain of salt. Nevertheless, we emphasize that the high-amplitude case responsible for a high correlation does not constitute a classical outlier, as the high tremor amplitude was indeed observed.

In the literature, there are a couple of studies that also put effort into analyzing hand movement using smartwatches [9,10,11] and smartphones [12,13,14]. These methods have already proved that using wireless devices could accelerate the data recording process and ease of access. However, there still exists physical contact with the user or patient in these methods, and using a contactless method provides more comfort and hygiene. Thus, studies on video-based analysis of hand movements/tremors have gained momentum recently. In one of them, Uhríková et al. [17] created signals using intensity changes from video frames. In some of the cases, they had errors over 1 Hz. In another study, Pintea et al. [18] used two different approaches to estimate the tremor frequency from the videos and had a 2.398 (±2.024) Hz Mean Average Error (MAE). In 2021, Williams et al. [20] employed videos recorded by a smartphone and, in most cases, achieved errors less than 0.5 Hz.

In our work, different from other video-based analysis studies, we focused on analyzing the effect of medication and aimed to do this by an automated approach achieving an accurate estimation of hand movement/tremor frequency from the video. Based on the results of the other papers, it is obvious that researchers have already achieved small errors for the tremor frequency estimation, and our results are also within that range. In contrast to our work, Uhríková et al. [17] and Williams et al. [20] used manual hand region of interest selection to conduct their study, which is a time-consuming method, while Pintea et al. [18] trained human body pose estimation models which also requires more time. In that sense, our work achieved an automatic estimation of hand movement/tremor frequency with a low error rate.

In the second step, we investigated the effect of medication on movement/tremor frequency and amplitude. Table 5 shows that for each patient, there was a decrease in terms of frequency. Two patients (PAT 001 and PAT 009) showed a typical parkinsonian tremor of around 4 Hz. All other patients had no classic tremor, but a low-frequency movement at between 1–2 Hz was detectable. We assume this rhythmic movement to be pulse related, i.e., a ballistocardiographic artifact. The heart beats with 60 to 90 beats per minute (1–1.5 Hz) at rest and creates a rhythmic signal in the hands, detectable in both the accelerometer and the video data. This signal shows that our approach is very sensitive to detect different frequencies, whether it is Parkinsonian tremor with around 4 Hz or the heartbeat conducted as a subtle movement artifact into the hands with around 1–2 Hz. In general, the patients that have a frequency for the before-medication case over 1.5 Hz, had frequency changes around 0.5 Hz. In addition to the frequency change, all patients also experienced a decreased amplitude value. These results showed that the short-time effect of medication on movement and tremor could be quantified automatically by a video-based approach.

## 5. Conclusions

In this work, we performed the automatic analysis of hand tremors using a video-based approach. Our study was based on employing high-frequency videos and tracking the hand via AI-based technology. In the first step, we investigated the frequency and amplitude relationship between the data extracted from the video and accelerometer. Results of the first step showed that our video-based approach can identify the tremor frequency by a small error rate (mean AE = 0.229 ± 0.174 Hz) and amplitude with a high correlation. In the second step, we focused on quantifying the effect of taking standard oral treatments like L-Dopa and dopamine agonists on tremor frequency and amplitude on video recordings. Our results suggest that patients experienced decreases in mean frequency from 2.012 ± 1.385 Hz to 1.526 ± 1.007 Hz and in mean amplitude from 8.167 ± 15.687 a.u. to 4.033 ± 5.671. Overall, our approach provides contactless automated analysis. Using this approach on a broader scale could have a significant impact in several scenarios. For one, it could make objective quantification of tremor in a medical setting easier for patients as well as the medical personnel. For another, it would allow unobtrusive tremor analysis at home, which would empower patients to supervise the progression of their disease or their response to medication with high accuracy on a daily basis.

## Figures and Tables

**Table 1:** Extended patient information.

Patient	Age	Sex	Years Since Disease Onset	L-Dopa Equivalent Doses (mg)		UPDRS * Part III (3.15)	H&Y *
				Daily	Before the Second Measurement		
PAT001	58	m	7	500	100	22 (2)	2
PAT002	83	f	13	632	133	35 (1)	3
PAT003	69	f	3	175	75	29 (0)	3
PAT004	67	m	7	600	100	26 (0)	2
PAT005	68	f	7	1125	150	36 (0)	2
PAT006	69	m	20	725	125	41 (1)	2–3
PAT007	52	m	17	785	75	26 (0)	2
PAT008	57	f	10	1716	133	48 (1)	2–3
PAT009	71	m	9	837	100	52 (3)	3
PAT010	63	m	10	601	109	73 (1)	3
PAT011	71	m	7	775	150	29 (0)	3

* UPDRS: Unified Parkinson’s Disease Rating Scale, Sum, (X) value of question 3.15, dominant side, * H&Y: Hoehn and Yahr.

**Table 2:** Descriptions of MediaPipe landmarks.

00 Wrist 01 Thumb carpometacarpal joint 02 Thumb metacarpophalangeal joint 03 Thumb interphalangeal joint 04 Thumb tip 05 Index finger metacarpophalangeal joint 06 Index finger proximal interphalangeal joint 07 Index finger distal interphalangeal joint 08 Index fingertip 09 Middle finger metacarpophalangeal joint 10 Middle finger proximal interphalangeal joint	11 Middle finger distal interphalangeal joint 12 Middle fingertip 13 Ring finger metacarpophalangeal joint 14 Ring finger proximal interphalangeal joint 15 Ring finger distal interphalangeal joint 16 Ring fingertip 17 Little finger metacarpophalangeal joint 18 Little finger proximal interphalangeal joint 19 Little finger distal interphalangeal joint 20 Little fingertip

**Table 3:** Dominant frequencies from the accelerometer and the video data.

Patient	Accelerometer (Hz)	Video Analysis (Hz)	AE (Hz)
PAT001	4.877	4.888	0.011
PAT002	1.001	1.223	0.222
PAT003	1.467	1.065	0.402
PAT004	1.533	2.087	0.554
PAT005	1.334	1.632	0.298
PAT006	1.258	1.054	0.204
PAT007	1.034	1.050	0.016
PAT008	1.577	1.638	0.061
PAT009	4.002	4.205	0.203
PAT010	1.134	1.462	0.328
PAT011	-	1.052	-
Mean	1.921 (±1.357)	1.941 (±1.338)	0.229 (±0.174)
RMSE	-	-	0.283

**Table 4:** Averaged amplitudes from the power spectrum. Accelerometer values from the video analysis are given in arbitrary units (a.u.).

Patient	Accelerometer (cm/s^2^)	Video Analysis (a.u.)
PAT001	14.251	5.720
PAT002	7.900	4.347
PAT003	12.589	2.213
PAT004	5.172	2.463
PAT005	4.7820	2.228
PAT006	3.2490	4.246
PAT007	6.259	1.982
PAT008	7.325	2.603
PAT009	202.873	52.637
PAT010	4.788	5.368
PAT011	n.a.	n.a.

**Table 5:** Estimated dominant frequencies.

Patient	Before Medication (Hz)	After Medication (Hz)	Frequency Change (Hz)
PAT001	4.888	3.447	−1.441
PAT003	1.065	1.024	−0.041
PAT004	2.087	1.027	−1.060
PAT005	1.632	1.030	−0.602
PAT006	1.054	1.027	−0.027
PAT007	1.050	1.028	−0.022
PAT008	1.638	1.217	−0.421
PAT009	4.205	3.421	−0.784
PAT010	1.462	1.025	−0.437
PAT011	1.052	1.017	−0.035
Mean	2.012 (±1.385)	1.526 (±1.007)	−0.487 (±0.491)

“−” sign means there is a frequency decrease.

**Table 6:** Averaged amplitudes for video data.

Patient	Before Medication (a.u.)	After Medication (a.u.)	Amplitude Change (a.u.)
PAT001	5.720	3.505	−2.215
PAT003	2.213	1.988	−0.225
PAT004	2.463	1.569	−0.894
PAT005	2.228	1.839	−0.389
PAT006	4.246	3.557	−0.689
PAT007	1.982	1.840	−0.142
PAT008	2.603	1.489	−1.114
PAT009	52.637	20.038	−32.599
PAT010	5.368	2.488	−2.880
PAT011	2.213	2.023	−1.900
Mean	8.167 (±15.687)	4.033 (±5.671)	−4.304 (±9.983)

“−” sign means there is an amplitude decrease.

## Data Availability

Not applicable.
